# An analysis of funding patterns in development assistance for mental health: who, when, what, and where

**DOI:** 10.1017/gmh.2020.30

**Published:** 2021-01-08

**Authors:** Rebecca S. F. Gribble, Bernhard H. Liese, Marisha N. Wickremsinhe

**Affiliations:** 1 Georgetown University, Washington, DC, USA; 2 University of Oxford, Oxford, UK

**Keywords:** Development assistance, International financing, Mental health

## Abstract

**Background:**

Mental health has recently gained increasing attention on global health and development agendas, including calls for an increase in international funding. Few studies have previously characterized official development assistance for mental health (DAMH) in a nuanced and differentiated manner in order to support future funding efforts.

**Methods:**

Data from the Organisation for Economic Cooperation and Development Creditor Reporting System were obtained through keyword searches. Projects were manually reviewed and categorized into projects dedicated entirely to mental health and projects that mention mental health (as one of many aims). Analysis of donor, recipient, and sector characteristics within and between categories was undertaken cumulatively and yearly.

**Findings:**

Between the two categories of official DAMH defined, characteristics differed in terms of largest donors, largest recipient countries and territories, and sector classification. However, across both categories there were clear and consistent findings: the top donors accounted for over 80% of all funding identified; the top recipients were predominantly conflict-affected countries and territories, or were receiving nations for conflict-affect refugees; and sector classification demonstrated shifting international development priorities and political drivers.

**Conclusion:**

Across DAMH, significant amounts of funding are directed toward conflict settings and relevant emergency response by a small majority of donors. Our analysis demonstrated that, within minimal international assistance for mental health overall, patterns of donor, recipient, and sector characteristics favor emergency conflict-affected settings. Calls for increased funding should be grounded in understanding of funding drivers and directed toward both emergency and general health settings.

## Introduction

Over the past 15 years, mental health has gained increasing attention on international agendas, including the declaration of mental health as both a global health and development priority (The Lancet, 2007; World Health Organization, 2008; World Health Organization, 2013; World Bank, 2016). This momentum continues through the Sustainable Development Goals era, with Goal 3 including a mental health specific indicator (United Nations, 2020*a*). Focus is finally shifting toward the long neglected and consistently increasing burden of disease attributable to mental, neurological, and substance abuse (MNS) conditions.

Over the past three decades, the disease burden of MNS conditions, including suicide, has risen globally, accounting for a total of 9.4% of all disability-adjusted life years in 2016 (Patel *et al*., 2018). Estimates suggest that mental disorders alone have accounted for 14% of age-standardized years lived with disability since the late 1990s, with a prevalence of over 10% in all global regions (GBD 2017 Disease and Injury Incidence and Prevalence Collaborators, 2018).

A key concern within the growing global attention on the burden of mental illness is awareness for the mental well-being of populations affected by conflict. The burden of some mental health disorders (depression, anxiety, post-traumatic stress disorder, bipolar disorder, and schizophrenia alone) in conflict-affected populations has an estimated prevalence of 22.1% at any point in time (Charlson *et al*., 2019). With an estimated 2 billion people currently living in fragile and conflict-affected areas, international organizations and media outlets have highlighted the significance of mental ill-health in these populations (International Organization for Migration, 2019; Percy, 2019; Save the Children, 2019; The Lancet, 2019; United Nations Office for the Coordination of Humanitarian Affairs, 2019).

This long standing ‘global health crisis’ calls for swift and internationally coordinated action (Patel *et al*., 2018). The need to act has been further amplified by the current COVID-19 pandemic, which may lead to increased need for mental health care across settings (United Nations, 2020*b*). Recent national surveys have reported high rates of psychological distress in general adult populations. For example, in the People's Republic of China, the percentage of respondents who reported psychological distress due to COVID-19 was 35%; in the United States, 45% of respondents indicated that their mental health has been negatively impacted by the pandemic; and in Iran, 61% of those surveyed reported to be in psychological distress (Jahanshahi *et al*., 2020; Panchal *et al*., 2020; Qiu *et al*., 2020). This initial body of evidence matches ongoing warnings from the global health field regarding the effects of COVID-19 and pandemic responses on the mental well-being of populations across the world (Bao *et al*., 2020; Dong and Bouey, 2020; Zandifar and Badrfam, 2020).

Amidst this ever-growing need for mental health services, a critical component is increased funding. Since 2015, five studies have sought to estimate development assistance for mental health (DAMH), identifying consistently low absolute values (Gilbert *et al*., 2015; Charlson *et al*., 2016; Turner *et al*., 2017; Lu *et al*., 2018; Liese *et al*., 2019). Despite application of varied criteria across different data sets and populations, all five studies arrived at the same conclusion: DAMH is equivalent to less than 1% of all development assistance for health (DAH). Two of these studies focused on funding for child and adolescent mental health, both finding that the humanitarian sector was the largest contributor to DAMH (Turner *et al*., 2017; Lu *et al*., 2018).

With a consensus on dismal international financing for mental health, we sought to conduct a nuanced analysis of DAMH across a decade using differentiated data. This more-detailed analysis allowed us to further refine our previous research, which demonstrated that just 0.3% of DAH was allocated to projects dedicated entirely to mental health between 2006 and 2016 (Liese *et al*., 2019). This paper systematically analyzes official DAMH from 2006 to 2016 to identify patterns in donors, recipients, and Organisation for Economic Cooperation and Development (OECD) sector categorization of DAMH. Analysis of funding patterns for mental health proves especially important given current calls for an international multi-sector financing partnership for investment in mental health (Patel *et al*., 2018).

## Methods

DAMH over the course of a decade was analyzed in detail using project-level data from the OECD Query Wizard for International Development (QWIDS) Database. Records were obtained in May 2018 following the method previously described (Organisation for Economic Co-operation and Development, 2018*a*; Liese *et al*., 2019). Analysis was performed in 2015 US dollars (constant prices).

All obtained projects were manually reviewed to ensure relevance to mental health and then classified into two categories based on the degree to which the project involved mental health. Development projects dedicated entirely to mental health are projects in which the entire project value can be ascertained to go toward mental health services, prevention, or awareness. Development projects that mention mental health include an element of mental health service, prevention or awareness amongst other non-mental health-related development activities, with only a portion of the total project value allocated toward mental health. See online Supplementary Tables S1 and S2 for a list of search terms used and examples of project categorization.

## Results

The OECD database search resulted in 9205 projects from 2006 to 2016 which included any use of a search term. Of the 9205 projects obtained, 1449 were eliminated due to project objectives not being relevant to mental health, leaving 7756 projects for analysis.

Through the manual review process, we noted a number of projects from the OECD categorized ‘Refugees in Donor Countries’ sector. The OECD recognizes official sector expenditure for refugees hosted in donor countries during the first 12 months of stay as official development assistance, therefore we removed these projects from the analysis (Organisation for Economic Co-operation and Development, 2018*b*). In total, 7734 projects equivalent to US$2451.0 million were included in the analysis as official DAMH as defined in this paper.

All projects retained for analysis were categorized to more accurately reflect global spending on mental health. Of the US$2451.0 million in DAMH from 2006 to 2016, just US$409.1 million (16.7%) was apportioned to projects dedicated entirely to mental health. The majority, US$2041.9 million (83.3%), was allotted to projects that mention mental health. The small proportion of DAMH for projects dedicated entirely to mental health was funded by 38 donors with assistance received by 136 countries and territories and 15 OECD regions. DAMH for projects that mention mental health alongside other development activities was funded by 44 donors with contributions received by 139 countries and territories and 15 OECD regions.

### DAMH donors

**Fig. 1.**
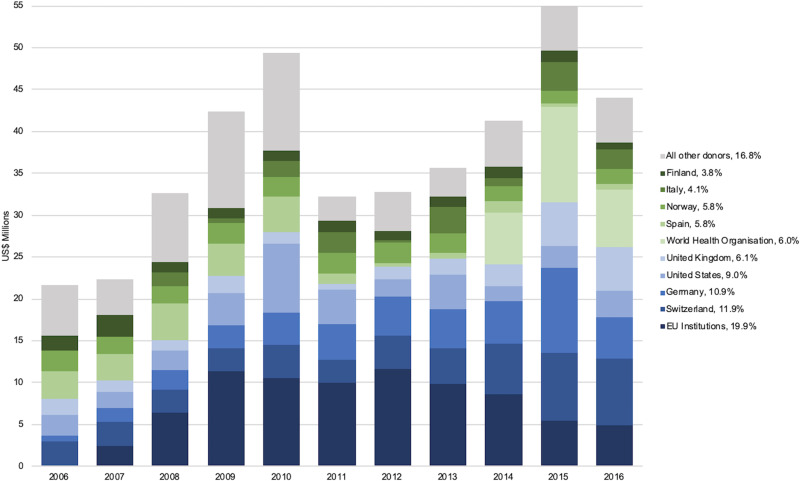
Ten largest donors for DAMH dedicated entirely to mental health by year, with a percentage of total DAMH dedicated entirely to mental health for 2006–2016 (in US$ millions).

Over half (57.8%) of all DAMH dedicated entirely to mental health across the decade analyzed was funded by just five donors: EU Institutions (US$81.3 million), Switzerland (US$48.7 million), Germany (US$44.7 million), the United States (US$36.7 million), and the United Kingdom (US$25.0 million). Switzerland, Germany, and the United Kingdom increased their DAMH dedicated entirely to mental health as the decade progressed, while the United States contributed their highest amount in 2010 (Fig. 1). The World Health Organization, Spain, Norway, Italy, and Finland were the sixth to tenth largest donors respectively, contributing an additional US$104.0 million (25.4%) across the decade.

**Fig. 2.**
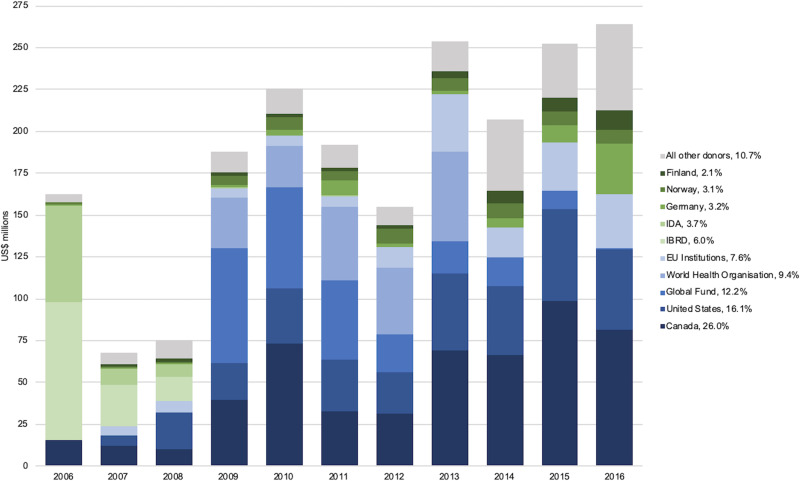
Ten largest donors for DAMH that mentions mental health by year, with a percentage of total DAMH that mentions mental health for 2006–2016 (in US$ millions). IDA, International Development Association; IBRD, International Bank for Reconstruction and Development.

The majority (71.2%) of DAMH for projects that mention mental health was funded by five main donors: Canada (US$531.5 million), the United States (US$327.9 million), the Global Fund (US$248.9 million), the World Health Organization (US$191.1 million), and EU Institutions (US$155.3 million). Canada increased contributions as the decade progressed alongside the United States and EU Institutions, while the Global Fund steadily decreased its contributions (Fig. 2). The International Bank for Reconstruction and Development, the International Development Association, Germany, Norway, and Finland were the sixth to tenth largest donors respectively for development projects that mention mental health, together contributing an additional US$368.3 million (18.0%) across the decade.

### DAMH recipients

**Fig. 3.**
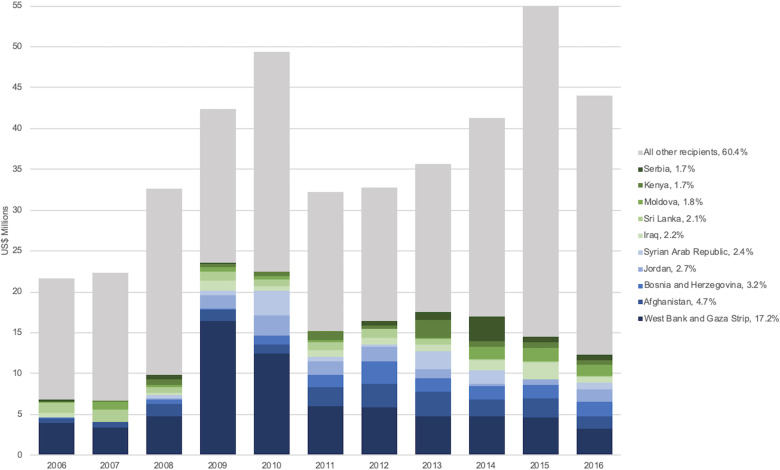
Ten largest recipients of DAMH dedicated entirely to mental health by year, with a percentage of total DAMH dedicated entirely to mental health for 2006–2016 (in US$ millions). *Note*: All other recipients include 142 additional countries, territories, regions, and unspecified recipients.

Of all recipients of DAMH dedicated entirely to mental health, the West Bank and Gaza Strip received the largest cumulative amount as well as the largest sum for every year analyzed. Development assistance received by the West Bank and Gaza Strip decreased markedly from 2009 (Fig. 3). The top 10 recipients of DAMH dedicated entirely to mental health across the decade analyzed were the West Bank and Gaza Strip (US$70.3 million), Afghanistan (US$19.3 million), Bosnia and Herzegovina (US$ 12.9 million), Jordan (US$11.2 million), Syrian Arab Republic (US$9.7 million), Iraq (US$8.9 million), Sri Lanka (US$8.8 million), Moldova (US$7.2 million), Kenya (US$7.2 million), and Serbia (US$6.8 million).

**Fig. 4.**
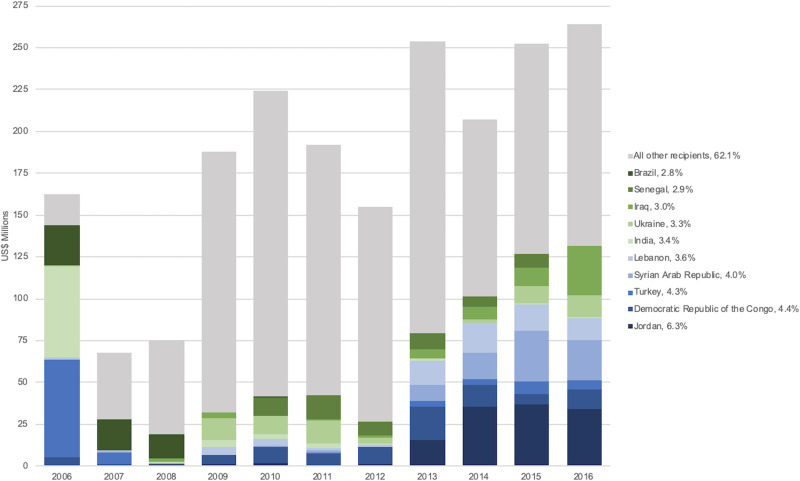
Ten largest recipients of DAMH that mentions mental health by year, with a percentage of total DAMH that mentions mental health for 2006–2016 (in US$ millions). *Note*: All other recipients include 145 additional countries, territories, regions, and unspecified recipients.

For DAMH that mentions mental health, Jordan was the largest recipient receiving a total of US$127.7 million, or 6.3% of this category from 2006 to 2016. Turkey, however, received the largest amount in a single year of US$58.3 million in 2006. As seen in Fig. 4, there is a clear increase in funding toward Jordan, the Syrian Arab Republic, Lebanon, and Turkey from 2013 onward. The top 10 recipients in this category were Jordan, Democratic Republic of the Congo (US$89.3 million), Turkey (US$86.8 million), Syrian Arab Republic (US$81.9 million), Lebanon (US$74.3 million), India (US$68.6 million), Ukraine (US$67.6 million), Iraq (US$61.4 million), Senegal (US$58.5 million), and Brazil (US$58.0 million).

### DAMH assistance categorization by OECD

**Fig. 5.**
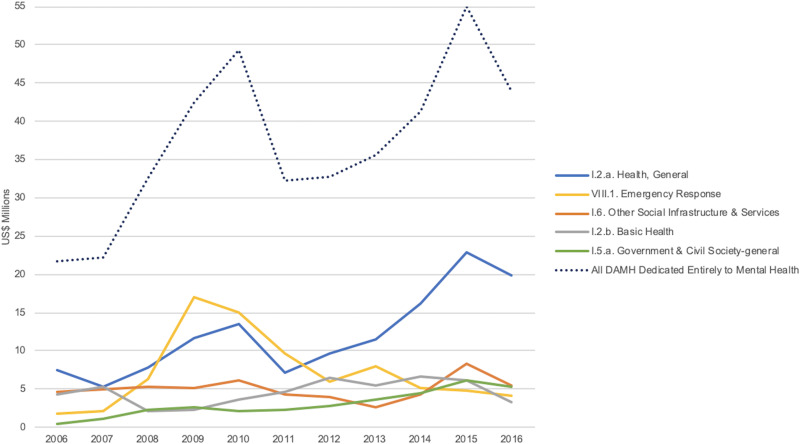
Five largest OECD sector categories for DAMH dedicated entirely to mental health and all DAMH dedicated entirely to mental health by year, in US$ millions.

Five OECD ODA sectors used to categorize DAMH dedicated entirely to mental health for 2006–2016 accounted for the majority (85.9%) of assistance. These top five sectors are illustrated in Fig. 5, with health sectors (‘Health, General’ and ‘Basic Health’) the largest category for all years analyzed except for 2009–2011 when more assistance was categorized as ‘Emergency Response’ sector.

**Fig. 6.**
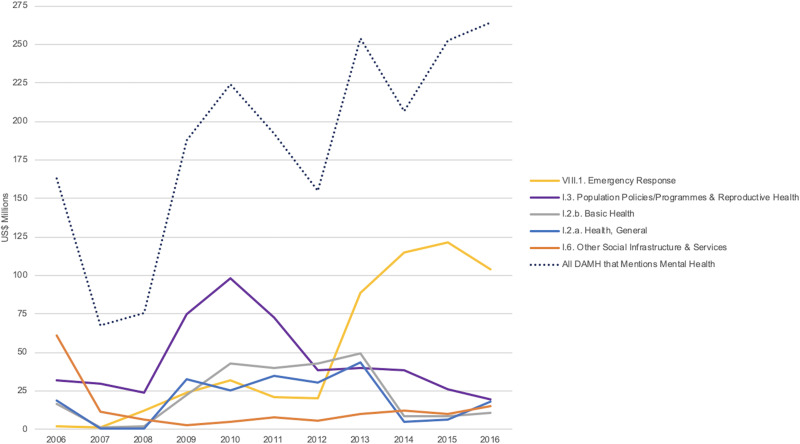
Five largest OECD sector categories for DAMH that mentions mental health and all DAMH that mentions mental health by year, in US$ millions.

Five OECD ODA sectors used to categorize DAMH that mentions mental health from 2006 to 2016 accounted for the majority (80.4%) of assistance. These top five sectors are illustrated in Fig. 6, with the largest amounts of assistance categorized as ‘Emergency Response’ or ‘Population Policies/Programmes & Reproductive Health’ sector assistance. There is a clear shift from ‘Population Policies/Programmes & Reproductive Health’ sector categorization to ‘Emergency Response’ sector categorization as the decade progressed.

## Discussion

The results obtained clearly demonstrate how official DAMH is intrinsically tied to, and influenced by, global political actions; as illustrated by OECD sector categorization and recipient countries and territories. For DAMH that mentions mental health, the highest amounts of assistance were categorized by the OECD as ‘Population Policies/Programmes & Reproductive Health’ sector or ‘Emergency Response’ sector assistance. From 2006 to 2012, the ‘Population Policies/Programmes & Reproductive Health’ sector was the largest OECD categorization of DAMH that mentions mental health. Over these 7 years, the ‘Population Policies/Programmes & Reproductive Health’ sector accounted for over one-third of all DAMH than mentions mental health, with almost all of this assistance (93.6%) tied to populations with or at risk of HIV/AIDS, meaning significant amounts of assistance during this time was for a specific global sub-population based on donor priorities. With just a few donors providing the majority of DAMH, OECD sector categorization appears representative of donor agendas.

Across both categories, DAMH is predominantly directed toward emergency or conflict settings. Although not all recipients are emergency settings or conflict-affected, the five largest recipients in both categories have experienced these situations or are receiving nations for refugees displaced within their region. This is evident in cumulative data and yearly data, with increases in funding received by recipients corresponding with conflict-related events (The New Humanitarian, 2020; United Nations, 2020*c*). This is indicated by the rise of DAMH dedicated entirely to mental health received by the West Bank and Gaza Strip in 2009, the increase in DAMH that mentions mental health received by the Syrian Arab Republic, Jordan, Turkey, and Lebanon from 2013 onward, and the increase in DAMH that mentions mental health categorized by the OECD as ‘Emergency Response’ sector assistance from 2013 onward.

Our analysis demonstrates that a significant amount of international funding for mental health is focused on a specific kind of population: those who are affected by conflict. Although a reassuring finding, and a critical use of DAMH, this must be considered within the context of meager overall amounts of international funding. Our previous study demonstrated that just 0.3% of ODA for health from 2006 to 2016 was allocated to projects dedicated entirely to mental health (Liese *et al*., 2019). And when including projects that mention mental health, 1.7% of ODA for health was directed toward mental health over the decade-long period (when applying entire project value, therefore an overestimation) (Liese *et al*., 2019). These small orders of magnitude mean no population – conflict-affected or not – receive sufficient funding.

In 2016, the countries and territories listed as the five largest recipients in both categories received varying amounts of official DAH, ranging from 0.1% to 36.0% of their total health spending (Global Burden of Disease Health Financing Collaborator Network, 2019). For Afghanistan and the Democratic Republic of the Congo, where development assistance accounts for 9.7% and 36.0% of total health spending respectively, donor behavior can have a large influence on health priorities and addressing population needs (Global Burden of Disease Health Financing Collaborator Network, 2019). For other countries, such as the Syrian Arab Republic and Jordan, official development assistance makes up just 1.5% and 2.1% of their total health spending respectively (Global Burden of Disease Health Financing Collaborator Network, 2019). Responsibility for financing population health issues, such as mental health disorders experienced by the conflict-affected, is then placed on national governments or individuals.

By categorizing DAMH into two categories – projects that are dedicated entirely to mental health and projects that mention mental health – we have been able to conduct an in-depth comparison and analysis to better understand international assistance for mental health. This analysis also allowed us to observe interrelated patterns between donor behaviors, recipients, and OECD sector categorization. Most notable is the sharp rise seen in DAMH dedicated entirely to mental health contributed by EU Institutions in 2009. This correlates to assistance received by the West Bank and Gaza Strip through the ‘Emergency Response’ sector that year (see Figs. 1, 3 and 5).

Analysis between projects dedicated entirely to mental health and projects that mention mental health shows that patterns of project data differ significantly across these two categories. If we analyzed all projects as an aggregate, we would capture only the dominant patterns from projects that mention mental health, as these projects constitute a much larger proportion of the funding. However, for projects that mention mental health, only a portion – of unknown value – of the total project budget is applied to mental health interventions. Results for DAMH that mentions mental health or analysis on the basis of mentioning mental health will overestimate, potentially quite significantly, the actual dollars contributed to mental health globally. Furthermore, an aggregated analysis of projects dedicated entirely to mental health and those that mention mental health will inevitably mask differences in funding patterns between these categories.

Consideration must also be given to the additional mental health needs of populations affected by the COVID-19 pandemic. Across conflict- and non-conflict-affected, low- and middle-income countries, responses for pandemic-related mental healthcare will be hampered by the historical underinvestment in mental health (United Nations, 2020*b*). In light of the many strains on healthcare systems to adequately provide mental healthcare, we call on the international community to increase sustainable funding for mental health across global populations.

Limitations of this paper's analysis include the use of a single data source, the OECD database. The use of this database restricts the analysis to OECD disbursements thereby excluding funding provided by international non-governmental organizations and private philanthropic donors (as this funding is not commonly captured in the database). Additionally, the database provides varying amounts of information at the project-level: some projects gave detailed descriptions while others offered only a few words. Therefore, the author's best judgment was used to determine both the relevance of projects to mental health and the degree to which projects involved mental health, which may impact project categorization in this analysis.

## Conclusion

Calls for the prioritization of mental health within development assistance and for significant increases in funding are present in the global health community. These much-needed calls to action must be considered alongside historical DAMH funding patterns. Our analysis identified that the majority of funding for mental health is politically driven, by being reactive to conflict settings and driven by donor agendas. A detailed analysis of past patterns, as provided by this paper, can open the discussion to where future funding should be allocated in consideration of recipient countries and territories and OECD sectors. Future funding for mental health must not only increase but be underpinned by a diversity of funding across settings.

## Supplementary material

For supplementary material accompanying this paper visit http://dx.doi.org/10.1017/gmh.2020.30. click here to view supplementary material
