# Supplementary material for: An analysis of funding patterns in development assistance for mental health: who, when, what, and where
Source: Glob Ment Health (Camb). 2021 Jan 8;8:e1. doi: 10.1017/gmh.2020.30 (PMC8057426; doi:10.1017/gmh.2020.30)
Supplement: Supplementary file 1 [file S2054425120000308sup001.zip › Supplementary Table 1.docx]

Supplementary Table 1: Search Terms Used.

| *Search terms used by Liese et al:* | |
| --- | --- |
| Addiction | Hypochondriasis |
| Affective | Insomnia |
| Alcohol | Learning disability |
| Antidepressant | Mania |
| Antipsychotic | Mental |
| Anxiety | Mood |
| Anxiolytic | Neurological |
| Autism | Personality disorder |
| Behavioral therapy | Phobia |
| Bipolar | Psychiatry |
| Cognitive impairment | Psychiatric |
| Delirium | Psychological |
| Delusion | Psychotic |
| Dementia | Psychosis |
| Dependency | Psychoses |
| Depression | Psychosocial |
| Depressive | Schizophrenia |
| Developmental disorder | Schizophrenic |
| Downs syndrome | Self harm |
| Dyslexia | Sleep disorder |
| Eating disorder | Somatoform |
| Electroconvulsive therapy | Stress disorder |
| Epilepsy | Substance abuse |
| Epileptic | Suicide |
| Hallucination | Trauma |
| Hyperactivity |  |
