# Supplementary material for: An analysis of funding patterns in development assistance for mental health: who, when, what, and where
Source: Glob Ment Health (Camb). 2021 Jan 8;8:e1. doi: 10.1017/gmh.2020.30 (PMC8057426; doi:10.1017/gmh.2020.30)
Supplement: Supplementary file 1 [file S2054425120000308sup001.zip › Supplementary Table 2.docx]

Supplementary Table 2: Examples of Project Categorization.

| *Category* | *Project example* |
| --- | --- |
| Project dedicated entirely to mental health | *Project Title “Psychosocial support for women/children in Eastern Chad”* |
| Project that mention mental health | Long Description: *“To provide consistent, appropriate, timely and accessible medical and psychological services to survivors and raise awareness of gender based violence and legislative and community action to combat it”*. |
| Project removed from analysis as not relevant to mental health due to alternate use of a search term | Search term: depression  Project Title: “*Reconstruction Efforts After the Tropical Depression Stan Disaster”* |
