# Supplementary figures and images for: An analysis of funding patterns in development assistance for mental health: who, when, what, and where
Source: Glob Ment Health (Camb). 2021 Jan 8;8:e1. doi: 10.1017/gmh.2020.30 (PMC8057426; doi:10.1017/gmh.2020.30)

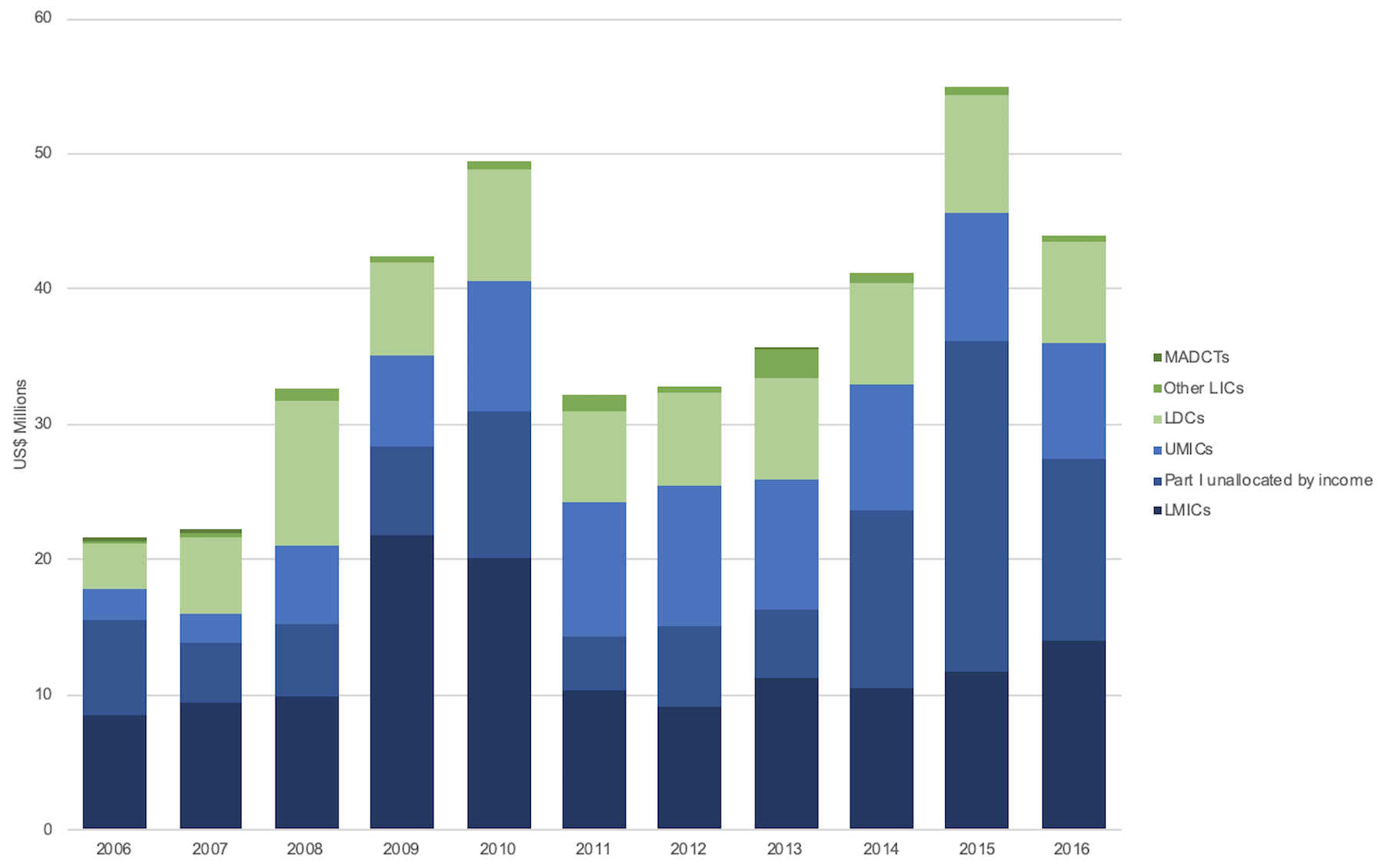

Supplement: Supplementary file 1 [file S2054425120000308sup001.zip › Supplementary Figure 1 LZW.tiff]

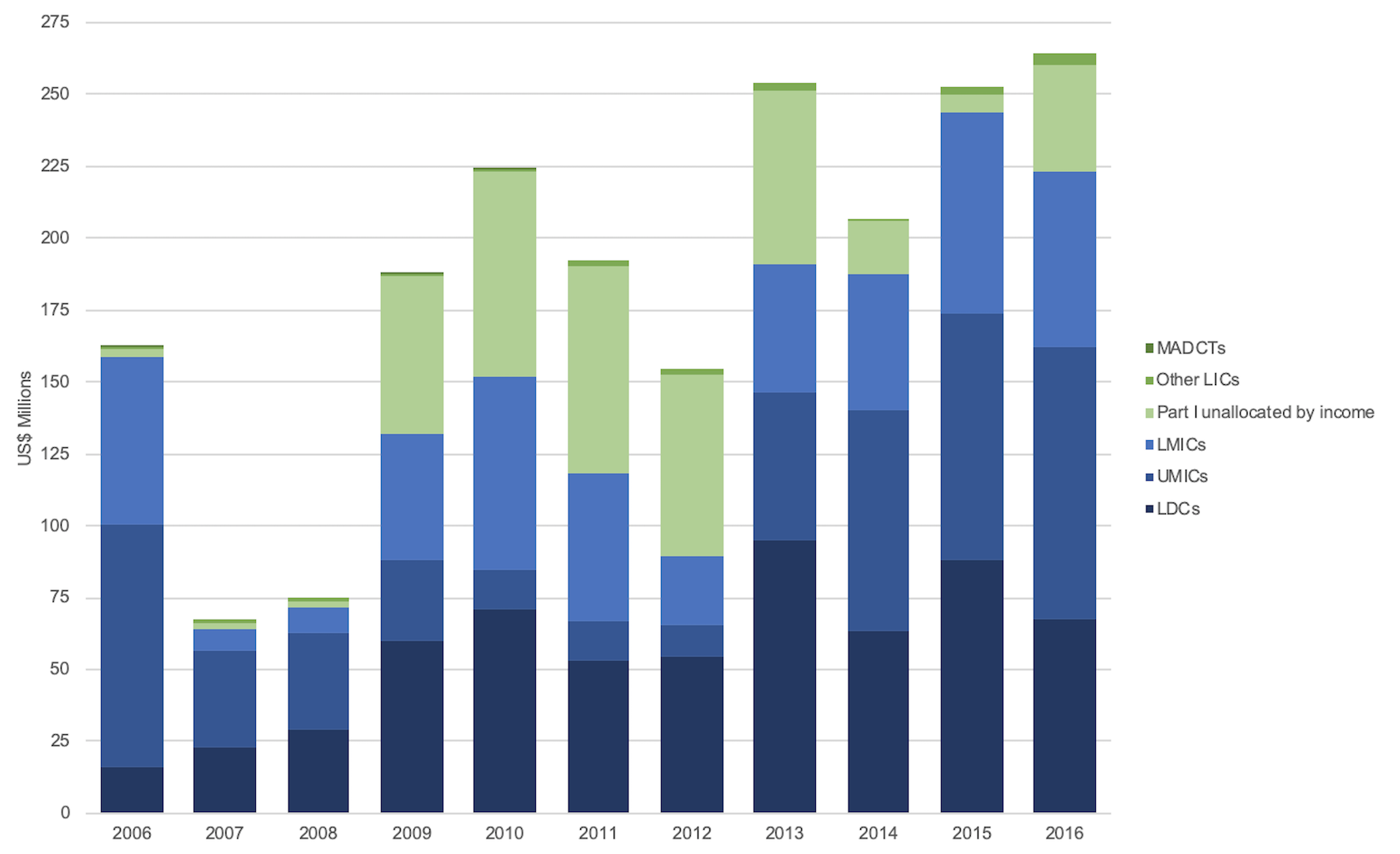

Supplement: Supplementary file 1 [file S2054425120000308sup001.zip › Supplementary Figure 2 LZW.tiff]

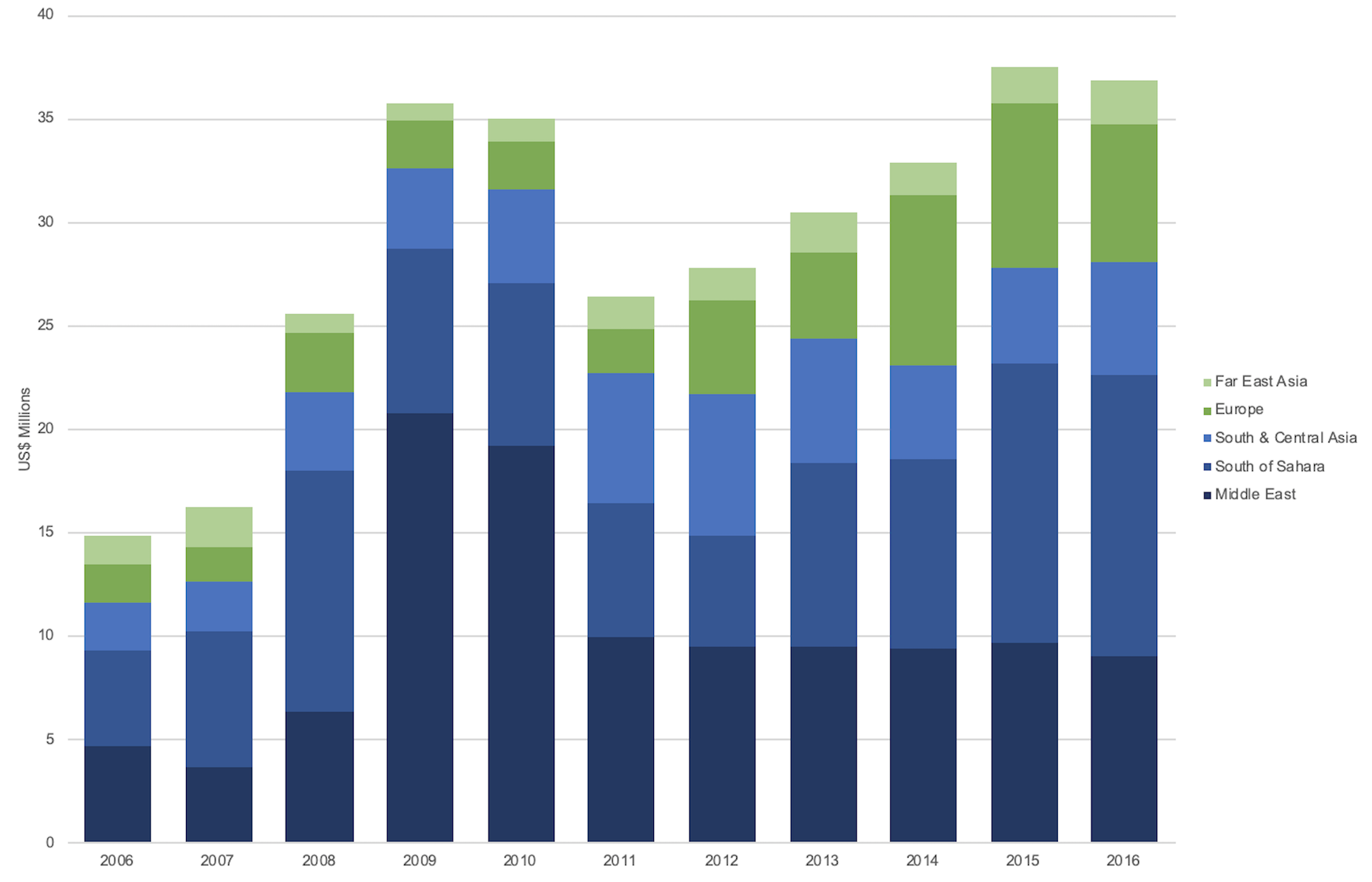

Supplement: Supplementary file 1 [file S2054425120000308sup001.zip › Supplementary Figure 3 LZW.tiff]

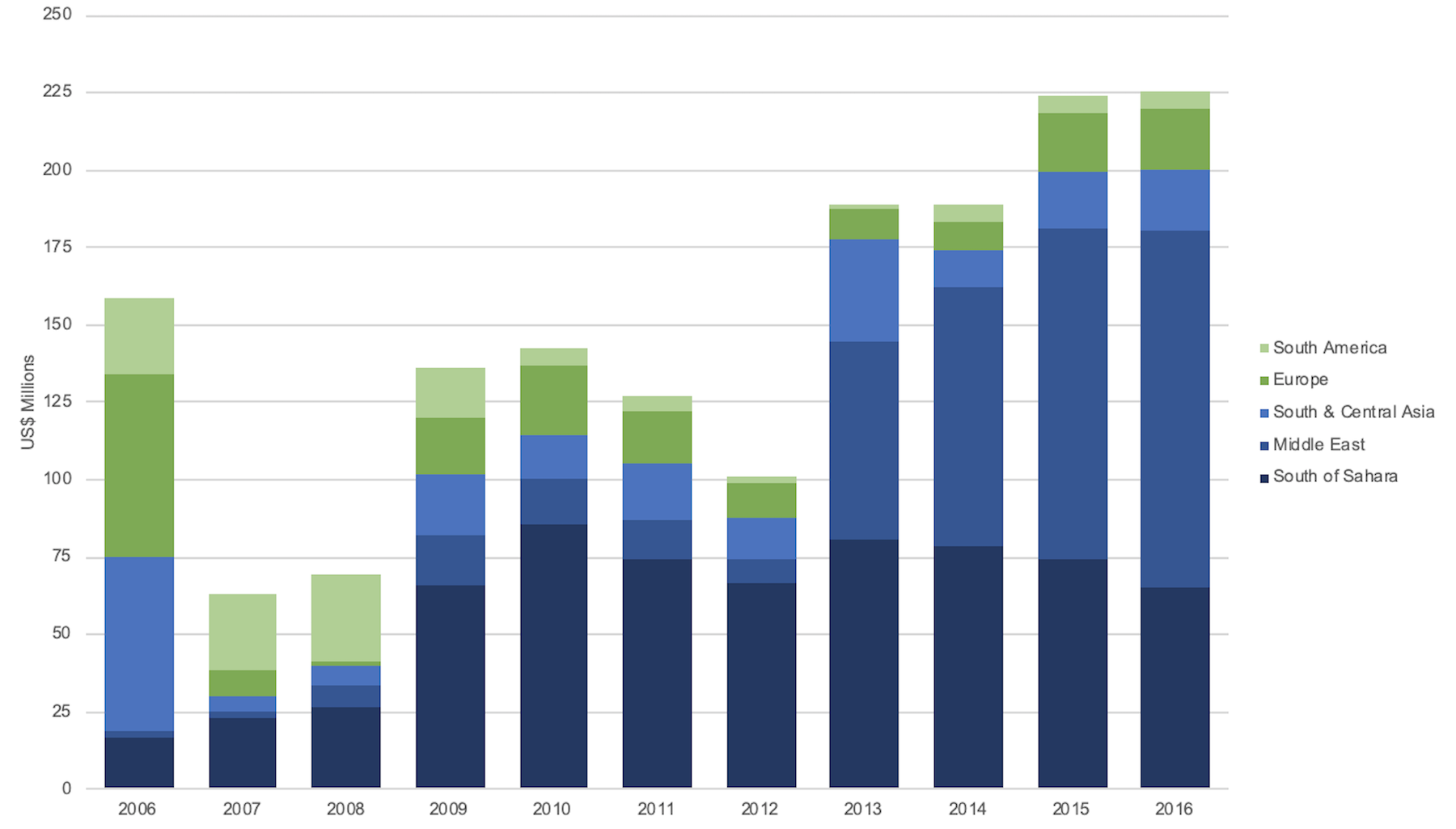

Supplement: Supplementary file 1 [file S2054425120000308sup001.zip › Supplementary Figure 4 LZW.tiff]
